# Supplementary material for: Sensitivity and robustness of sky-polarimetric Viking navigation: Sailing success is most sensitive to night sailing, navigation periodicity and sailing date, but robust against weather conditions
Source: PLoS One. 2022 Feb 2;17(2):e0262762. doi: 10.1371/journal.pone.0262762 (PMC8809617; doi:10.1371/journal.pone.0262762)
Supplement: Attachment — Submitted filename: +VikingNavigation_PLoS-One_response.docx [file pone.0262762.s001.docx]

**Point-by-Point Response to the Comments of Referee 1**

We thank the positive and constructive review of Referee 1. Our manuscript was revised on the basis of the reports of two Reviewers. All changes suggested by Referee 1, Referee 2 and the Editor are marked with green, orange and blue, respectively. Below is our detailed Point-by-Point Response to the comments of Referee 1.

**Referee 1 wrote**: *There are some very minor issues with grammar and style, but to improve the readability of the ms I suggest the authors go over their ms once again carefully, preferably with someone with an excellent command of English unless the journal assists in this matter. I am giving some example of some minor corrections below:*

*Line 27: "did not have magnetic compasses" or "did not have a magnetic compass"*

*L 28: "under a sunny sky"*

*L37: "which had a strong and a weak influence" or "which had strong and weak influences.."*

*L40: "while it is robust"*

*L42: "and changing cloudiness" or "..changing cloud cover.."*

*L51: "without a magnetic compass..!"*

*L52:"under sunny weather conditions"*

*L56: delete "their"*

*L82. "have a strong and weak"*

**Answer**: All these grammatical/stylistical corrections are performed in our revised manuscript.

**Referee 1 wrote**: *1. Why were the voyages simulated only at spring equinox and summer solstice? What about the other days of the year?*

**Answer**: To the revised subsection ‘Computational methods’ we added the following paragraph:

The spring equinox (21 March) and the summer solstice (21 June) meant two important dates of the Viking sailing season: the former and latter was approximately the start and the middle of this season. In our simulations we chose only the spring equinox and the summer solstice as sailing dates, because the two artefacts of the Viking sun-compass (discovered in the Vatnahverfi and Uunartoq Fjords of Greenland) had only two clearly discernible gnomonic lines (trajectories of the tip of the shadow cast by the vertical gnomon on the horizontal disc of the sun-compass in sunshine) [3]: a straight line for navigation at spring equinoxes and a hyperbolic line for summer solstices. For other sailing dates the gnomonic lines are different hyperbolic curves.

**Referee 1 wrote**: *2. The authors wrote: The simulated Viking ship moved in a constant direction along a straight line with a constant (but varying) speed...*

*How can the speed be constant, if it is varying? Please clarify this.*

**Answer**: The criticized sentences were reworded as follows:

During a given time period τ, the simulated Viking ship moved in a constant direction along a straight line with a constant sailing speed *w* until the navigator determined the new sailing direction. In the simulation, the time segment τ was chosen randomly with a uniform distribution from the interval Δ*t* - Δ*t*/6 ≤ τ ≤ Δ*t* + Δ*t*/6, where Δ*t* is the navigation periodicity (= the elapsed time since the last navigation) [34].

**Referee 1 wrote**: *3. The authors wrote: The angle of deviation from the geographical north, called north error, was determined as described in [34].*

*Please summarize briefly this determination in order that the readers do not need to consult with article [34].*

**Answer**: The subsection ‘*North error*’ of the ‘Methods’ section was revised in the following way:

The angle of deviation from the geographical north, called north error, was determined as follows: For a given solar elevation angle θ we calculated the actual cloudiness ρ as described above, then we used the 1080 data files from the θ-ρ matrix determined in [31]. In this matrix, every θ-ρ pair contains 12 different sky situations with a known north error distribution calculated with the use of the errors of the four steps of SPVN measured in psychophysical experiments. One of these 12 sky situations was selected randomly according to uniform distribution, then from the corresponding error distribution we used a randomly chosen error value as the actual north error.

**Referee 1 wrote**: *4. Do the authors plan to continue their study with such a computer simulation in which they compare the success of Viking navigation when the navigator takes into consideration not only the sky polarization, but also or only the Sun, if it is visible? This would be important, because during a several-week Viking voyage the Sun was surely visible for many occasions, thus in sunshine the navigator did not need to use the polarization pattern of the sky. This possibility should briefly be addressed in the Discussion section.*

**Answer**: In a separate paper we plan to study the sky-polarimetric Viking navigation so that we compare the success of Viking navigation when the navigator takes into consideration not only the sky polarization, but also or only the Sun, if it is visible. Such a simulation is important, because during a several-week Viking voyage the Sun was surely visible for many occasions, thus in sunshine the navigator did not need to use the polarization pattern of the sky.

**Referee 1 wrote**: *5. Would it have made much difference if the departure had not been from Bergen, but further north or south in Norway? Was that because of prevailing ocean currents that were taken into consideration?*

**Answer**: To the revised subsection ‘Computational methods’ we added the following paragraph:

The route of the Viking voyage between Norway and south Greenland (Hvarf) ran along the 60° 21' 55'' northern latitude and started from Hernam (nowadays the Norwegian Bergen) [2, 3]. One of the possible explanations of this latitude could be that the prevailing ocean currents might have been taken into consideration by Vikings. On the other hand, this straight route was the shortest between Norway and Hvarf. If the departure had not been from Bergen, but much further north or south in Norway, then the duration of a voyage would have been longer, and other shapes of the gnomonic lines should have been used as the straight (for spring equinoxes) or hyperbolic (for summer solstices) lines found on the sun-compass artefacts.

**Point-by-Point Response to the Comments of Referee 2**

We thank the constructive review of Referee 2. Our manuscript was revised on the basis of the reports of two Reviewers. All changes suggested by Referee 1, Referee 2 and the Editor are marked with green, orange and blue, respectively. Below is our detailed Point-by-Point Response to the comments of Referee 2.

**Referee 2 wrote**: *The paper represents an important sequel to previous studies done by the authors in the topic of sky-polarimetric Viking navigation. It provides further elaboration on the potential success rates of sailing to the shores of Greenland from Norway by using a polarization-based navigation technique with dichroic crystals.*

*More specifically, here the authors present their findings obtained via a simulation model that has been refined compared to its original version used in their former study. This new model has more subtle features, and as such, it is capable of providing a more accurate representation of relevant factors, including weather, that may influence the success of polarization-based sailing.*

*One of the main finding of the paper is that navigation by polarization can be very robust against weather conditions, which could be a fundamental factor in verifying the operability and veracity of sky-polarimetric Viking navigation.*

**Answer**: We thank Referee 2 for this nice summary. To the revised Discussion we added the following introducing paragraph:

This work is a logical sequel of our previous investigation [34] in the topic of sky-polarimetric Viking navigation (SPVN). We provide a further elaboration of the potential success rate of sailing by SPVN using dichroic/double-refracting sunstone crystals to the southeast shore of Greenland from Norway. Our new findings are obtained via a computer simulation which is refined compared to its original version [34]. This improved model has subtler features, and therefore, is capable to take into consideration more precisely also the weather that may influence the success of SPVN. One of the main findings of this paper is that SPVN can be very robust against weather conditions, which could be a fundamental factor in verifying the operability and veracity of this navigational method.

**Referee 2 wrote**: *1. Pg. 3., lines 142-149: "Each succeeding ρ-value was calculated by adding a discrete change (increment) in okta to the previous ρ-value according to a second Gaussian distribution with median m_dominance_ and deviation σ_changeability_,..."*

*The authors here describe how the cloudiness is set and changed along a given sailing route. One of the introduced parameters is cloudiness dominance m_dominance_. If I understand well, then m_dominance_ solely drives the increment or decrement of the ρ-value (okta) in skewing the change of ρ-values towards more or less negative or positive changes. However, in order that this scheme work without automatically converging to and then getting stuck at zero cloudiness or total cloudiness, σ_changeability_ is needed (with a sufficiently greater range of values than those of m_dominance_) to allow variation to the preset cloudiness dominance.*

*In my opinion this should be explained somewhat in more detail in the paper so that it be completely clear what are the consequences of using these particular parameters and their available ranges. In fact, it might be even better to check and see that along a given route what is the average and deviation of ρ-values when using a given m_dominance_ and σ_changeability_, and among all simulated routes what is their distribution.*

**Answer**: As suggesed, to the revised ‘*Discussion*’ the following was added:

In our simulations, variable *m*_dominance_ drives the increment/decrement of cloudiness ρ (okta) in skewing the change of ρ-values towards more or less negative or positive changes. In order that this scheme can work without automatically converging to and then getting stuck at zero cloudiness (ρ = 0 okta) or total cloudiness (ρ = 8 okta), variable σ_changeability_ is needed (with an appropriately greater range of values than that of *m*_dominance_) to allow variation to the preset cloudiness dominance. As examples, Fig. 8 shows the cloudiness distributions during simulated voyages using cloudiness dominances *m*_dominance_ = -1, 0 or +1 (meaning dominantly weakly, medium or strongly cloudy skies) and cloudiness changeabilities σ_changeability_ = 1, 2 or 4 (meaning weakly/slowly, medium or strongly/rapidly changing cloudiness).

**Fig. 8. Cloudiness distributions of Viking sailing routes.** Cloudiness distributions during simulated voyages using calcite sunstones at summer solstice with navigation periodicity Δ*t* = 1 hour for cloudiness dominances *m*_dominance_ = -1, 0 or 1 (meaning dominantly weakly, medium or strongly cloudy skies) and for cloudiness changeabilities σ_changeability_ = 1, 2 or 4 (meaning weakly/slowly, medium or strongly/rapidly changing cloudiness).

**Referee 2 wrote**: *2. Pg. 4. lines 153-154: "The simulated Viking ship moved in a constant direction along a straight line with a constant (but varying) speed until ["]"*

*This sentence would require some rewording, as "constant (but varying) speed" is a contradictory statement. I suppose what is meant here is that sailing speed was constant during a given time period, but across time segments it could be varied.*

**Answer**: The criticized sentences were reworded as follows:

During a given time period τ, the simulated Viking ship moved in a constant direction along a straight line with a constant sailing speed *w* until the navigator determined the new sailing direction. In the simulation, the time segment τ was chosen randomly with a uniform distribution from the interval Δ*t* - Δ*t*/6 ≤ τ ≤ Δ*t* + Δ*t*/6, where Δ*t* is the navigation periodicity (= the elapsed time since the last navigation) [34].

**Referee 2 wrote**: *3. Fig. 2B and Fig. 4-6.: It is striking that the success rates of simulated sailing routes are practically independent of cloudiness dominance 'm_dominance_', as it is also described by the authors in the Results and Discussion parts. However, this would warrant some deeper explanation in my view. I would assume that this has more or less something to do with the cumulated navigation error due to cloudiness having such a low scatter in the position of the sailing route end point so that it practically does not change the success rate in the definition of the paper's model. This would be worth checking against, and it might be also interesting to see a scatter plot of sailing routes (or only their end points) at a given 'm_dominance_' value (or in a restricted range).*

**Answer**: To the revised ‘*Discussion*’ section we added the following:

It is striking that the success rate *s* of simulated sailing routes are practically independent of the dominance *m*_dominance_ and changeability σ_changeability_ of cloudiness (Figs. 2B, 4-6). This can partly be explained with the cumulated navigation (north) error due to cloudiness having only a low scatter near the end point of the sailing route so that it practically does not change the success rate *s*. Note that *s* depends mainly on the (a)symmetry of the particular north error cumulated in the morning and the afternoon of every day. Using the same data series as we in this work, Száz et al. [31] have already studied the dependence of the north error on the cloudiness ρ, taking into consideration the solar elevation and the sailing date (spring equinox or summer solstice). They also found (see Fig. 4 of [31]) that both the net (cumulated) morning and the net afternoon north errors are practically independent of ρ, but depend on the sailing date (spring equinox or summer solstice), the solar elevation and the time of day (morning or afternoon). Hence, since the north error does not vary significantly with changing ρ, ρ has no great effect on the morning-afternoon asymmetry, and therefore it influences only slightly the success rate *s* of SPVN.

**Referee 2 wrote**: *3. Here I would also propose an aspect that may be out of scope of this paper but perhaps relevant to some future work: the evaluation of simulation results could include a progressive restriction of the coast line section accepted as successful destination. It could be studied how far this can be restricted (i.e. the desired destination to be more and more localized) and how success rates degrade depending on relevant factors.*

**Answer**: To the revised ‘*Discussion*’ we added the following paragraph:

In the future, it could be studied how a progressive restriction of the coastline section defined as successful destination influences the success rate *s* of Viking navigation. It is expected that *s* gradually decreases as the desired destination becomes more and more localized. Thus, the chance of a precise localization of the destination is small in all probability.

**Referee 2 wrote**: *4. Fig. 3B,D: There is a clear inversion of the success rate trend against navigation periodicity Δt between spring equinox and summer solstice, when allowing night sailing. Is there anything that can be said how this would look like at other dates and if there were any periods of the year, when a proper navigation periodicity could be selected for such a sailing mode?*

**Answer**: To the revised ‘Results’ section we added the following:

Without night sailing, the navigational success rate *s* tendentiously, but with oscillation, decreases with increasing navigational period Δ*t* at both spring equinox and summer solstice (Fig. 3A,C). On the other hand, With night sailing there is an inversion of the change of *s* versus Δ*t* between spring equinox and summer solstice (Fig. 3B,D): At spring equinox *s* tendentiously, oscillatory increases with increasing Δ*t* (Fig. 3B), while at summer solstice *s* tendentiously decreases with increasing Δ*t* (Fig. 3D). From these characteristics it follows that without night sailing it was worth using a navigational period Δ*t* = 1 hour in order to ensure high (80-100 %) success rate *s* at both spring equinox and summer solstice. However, with night sailing, if Viking navigators wanted always (at both spring equinox and summer solstice) high (80-100 %) *s*, they should navigate Δ*t* = 3-4 hourly.

Concerning other sailing dates than spring equinox and summer solstice, to the revised subsection ‘Computational methods’ we added the following paragraph:

The spring equinox (21 March) and the summer solstice (21 June) meant two important dates of the Viking sailing season: the former and latter was approximately the start and the middle of this season. In our simulations we chose only the spring equinox and the summer solstice as sailing dates, because the two artefacts of the Viking sun-compass (discovered in the Vatnahverfi and Uunartoq Fjords of Greenland) had only two clearly discernible gnomonic lines (trajectories of the tip of the shadow cast by the vertical gnomon on the horizontal disc of the sun-compass in sunshine) [3]: a straight line for navigation at spring equinoxes and a hyperbolic line for summer solstices. For other sailing dates the gnomonic lines are different hyperbolic curves.

**Referee 2 wrote**: *5. Fig. 2. and Fig. 3C,D: the graphs show high amplitude oscillation of success rates against navigation periodicity Δt for certain periods, with quite low and high local extrema. Can the authors explain what are the reasons of this? I suppose the explanation may partly be given by North error accumulations as displayed on Fig. 7, that is, with higher navigation periodicities than 1 hour, the absolute accumulated error can greatly vary, depending in which discrete points the actual navigation happens, so that for a certain periodicity the compensation of North accumulation error can be good, while for a slightly different periodicity it can break down quickly by accumulating error skewed into one of the directions.*

**Answer**: You are right, therefore, to the revised ‘*Discussion*’ we added the following paragraph:

The graphs in Figs. 2. and 3A-C show smaller- or higher-amplitude oscillation of the success rate *s* versus the navigation periodicity Δ*t* for certain periods with lower and higher local extrema. The main reason for this phenomenon is the alternating sign of the strongly oscillating north error accumulated on a day (Fig. 7): At higher navigation periodicities than 1 hour, the absolute accumulated north error can greatly vary, depending on the actual point of time of navigation. Thus, for a certain periodicity Δ*t* the compensation for the accumulated north error can be good, while for a slightly different Δ*t*-value it can break down quickly by accumulating north error skewed into one of northern or southern directions.

**Point-by-Point Response to the Editor**

We thank the positive and constructive review of Referee 1. Our manuscript was revised on the basis of the reports of two Reviewers. All changes suggested by Referee 1, Referee 2 and the Editor are marked with green, orange and blue, respectively. Below is our detailed Point-by-Point Response to the comments of the Editor.

**Editor wrote**: *1. Please ensure that your manuscript meets PLOS ONE's style requirements, including those for file naming. The PLOS ONE style templates can be found at*

*https://journals.plos.org/plosone/s/file?id=wjVg/PLOSOne_formatting_sample_main_body.pdf*

*and*

*https://journals.plos.org/plosone/s/file?id=ba62/PLOSOne_formatting_sample_title_authors_affiliations.pdf*

**Answer**: Our revised manuscript meets PLOS ONE's style requirements, including file naming.

**Editor wrote**: *2. Please review your reference list to ensure that it is complete and correct. If you have cited papers that have been retracted, please include the rationale for doing so in the manuscript text, or remove these references and replace them with relevant current references. Any changes to the reference list should be mentioned in the rebuttal letter that accompanies your revised manuscript. If you need to cite a retracted article, indicate the article's retracted status in the References list and also include a citation and full reference for the retraction notice.*

**Answer**: Our reference list is complete and correct, and it does not contain a retracted publication.

**Editor wrote**: *3. Please include in your Methods section details of how the model was implemented. If the code has been or can be publicly shared, please also include information on where the code can be found, both in the Methods section and the Data availability statement.*

**Answer**: In the revised ‘*Methods*’ section we gave further details about our simulation model, as required also by both Referees.

**Editor wrote**: *4. We note that Figure 1 in your submission contain map images which may be copyrighted. All PLOS content is published under the Creative Commons Attribution License (CC BY 4.0), which means that the manuscript, images, and Supporting Information files will be freely available online, and any third party is permitted to access, download, copy, distribute, and use these materials in any way, even commercially, with proper attribution. For these reasons, we cannot publish previously copyrighted maps or satellite images created using proprietary data, such as Google software (Google Maps, Street View, and Earth). For more information, see our copyright guidelines:*

*http://journals.plos.org/plosone/s/licenses-and-copyright*

*We require you to either (1) present written permission from the copyright holder to publish these figures specifically under the CC BY 4.0 license, or (2) remove the figures from your submission:*

*a. You may seek permission from the original copyright holder of Figure 1 to publish the content specifically under the CC BY 4.0 license.*

*We recommend that you contact the original copyright holder with the Content Permission Form*

*http://journals.plos.org/plosone/s/file?id=7c09/content-permission-form.pdf*

*and the following text: "I request permission for the open-access journal PLOS ONE to publish XXX under the Creative Commons Attribution License (CCAL) CC BY 4.0 (http://creativecommons.org/licenses/by/4.0/). Please be aware that this license allows unrestricted use and distribution, even commercially, by third parties. Please reply and provide explicit written permission to publish XXX under a CC BY license and complete the attached form."*

*Please upload the completed Content Permission Form or other proof of granted permissions as an "Other" file with your submission.*

*In the figure caption of the copyrighted figure, please include the following text: "Reprinted from [ref] under a CC BY license, with permission from [name of publisher], original copyright [original copyright year]."*

*b. If you are unable to obtain permission from the original copyright holder to publish these figures under the CC BY 4.0 license or if the copyright holder's requirements are incompatible with the CC BY 4.0 license, please either i) remove the figure or ii) supply a replacement figure that complies with the CC BY 4.0 license. Please check copyright information on all replacement figures and update the figure caption with source information. If applicable, please specify in the figure caption text when a figure is similar but not identical to the original image and is therefore for illustrative purposes only. The following resources for replacing copyrighted map figures may be helpful:*

*USGS National Map Viewer (public domain):*

*http://viewer.nationalmap.gov/viewer/*

*The Gateway to Astronaut Photography of Earth (public domain):*

*http://eol.jsc.nasa.gov/sseop/clickmap/*

*Maps at the CIA (public domain):*

*https://www.cia.gov/library/publications/the-world-factbook/index.html*

*and*

*https://www.cia.gov/library/publications/cia-maps-publications/index.html*

*NASA Earth Observatory (public domain): http://earthobservatory.nasa.gov/*

*Landsat: http://landsat.visibleearth.nasa.gov/*

*USGS EROS (Earth Resources Observatory and Science (EROS) Center) (public domain):*

*http://eros.usgs.gov/*

*Natural Earth (public domain): http://www.naturalearthdata.com/*

*[Note: HTML markup is below. Please do not edit.]*

**Answer**: We do not need a permission to publish the map in Figure 1, because to the subsection ‘*Visibility distance of Greenland’s southeast coastline (end of simulation)*’ we added the following:

The map of the North Atlantic region was generated by a software written by us. The contours of continents and islands were manually digitalized from the map available as open-source data from http://www.gnuplotting.org/plotting-the-world-revisited/ (the raw data points of the contours can be freely downloaded in text format from: http://www.gnuplotting.org/data/world_10m.txt). These open-source data can be freely used without permission/licence.

Furthermore, to the legend of Figure 1 we also added:

The maps are generated by our software after a manual selection of the contours of continents and islands from the open-source data originating from http://www.gnuplotting.org/plotting-the-world-revisited/.
